# Examining the predictors of healthcare facility choice for outpatient care among older adults in India using Andersen’s revised healthcare utilization framework model

**DOI:** 10.1186/s12877-022-03634-y

**Published:** 2022-12-09

**Authors:** Margubur Rahaman, Pradip Chouhan, Avijit Roy, Md. Juel Rana, Kailash Chandra Das

**Affiliations:** 1 Senior Research Fellow, Department of Migration & Urban Studies, International Institute for Population Sciences (IIPS), Mumbai, 400088 India; 2 Professor, Department of Geography, University of Gour Banga, Malda, 732103 India; 3 State Aided College Teacher, Department of Geography, Malda College, Malda, 732101 India; 4 Assistant Professor, Govind Ballabh Pant Social Science Institute (GBPSSI), Allahabad, 211019 India; 5 Professor, Department of Migration & Urban Studies, International Institute for Population Sciences (IIPS), Mumbai, 400088 India

**Keywords:** Outpatient care, Older adults, Healthcare responsiveness, Spatial patterns, India

## Abstract

**Background:**

In India, the demand for outpatient care is substantially higher than inpatient care among older adults. Therefore, the current study examines the level, patterns, and factors associated with outpatient care use.

**Methods:**

The present research used data from the first wave of the Longitudinal Ageing Study in India (LASI, 2017–18). A total of 34,588 older adults (45 years and above) who accessed outpatient healthcare services in one year prior to the survey were included in this study. A bivariate chi-square test was applied to present the percentage distribution of types of outpatient healthcare utilization by background characteristics and healthcare responsiveness. Multinomial logistic regression analyses were employed to explore the interplay of outpatient healthcare utilization and allied predisposing, enabling, and need factors.

**Results:**

About 63.7% of total older adults used a private facility, followed by 22.8% used a public facility, and 13.5% used other facilities. Years of schooling, household wealth status, place of residence, self-rated health, and health insurance were all found to be significant determinants of public or private facility use. In contrast, respondents' sex was found to be a significant determinant of private healthcare use only. The study finds that there was inadequate healthcare reaction to public health facilities.

**Conclusion:**

The current study revealed that the use of private facility for outpatient care is noticeably high in India. Older adults' educational attainments, health insurance coverage, and household level economic background were found to be significant factors in healthcare choice. The current study emphasizes the need to strengthen public healthcare services for outpatient care.

## Background

Globally, less developed regions have been experiencing faster growth in the aging population [1]. As a result, nearly 80% of the world's older population will reside in less developed regions by 2050. Regional patterns of population aging show the older population in the Asia–Pacific region has been increasing fast, and it is expected to reach 1.3 billion people by 2050 [1, 2]. Among the Asia–Pacific countries, India has been going through an unprecedented pace of population aging and is expected to reach 319 million by 2050, accounting for about 20% of the total population [3]. Therefore, the research attention has shifted greatly toward older adults' health, healthcare utilization, and social welfare in order to attain successful and healthy aging in India.

According to the most recent Indian census of 2011, the number of older people in India is 104 million [4], which underlines the high demand for the healthcare system because of the high prevalence of multi-morbidities among older adults [5]. The high prevalence of communicable diseases (CDs) like diarrhea (15%), malaria (9%), and typhoid (6%) among the older population is a well-known public health challenge in India [5]. The epidemiological shift toward non-communicable diseases (NCDs) in developing nations, including India, triggered public health service demand for both inpatient and outpatient care services [6]. The latest Longitudinal Ageing Study in India (LASI), 2017–18 report shows a substantial proportion of older adults (45 years and above) suffering from cardio-vascular-diseases (CVDs) (27.7%), hypertension (26%), diabetes (11%), and anemia (5%), asthma (4), and heart diseases (4%) [5]. As a result, the dual burden of CDs and NCDs among older adults prompted demand for outpatient healthcare services in India [6]. The LASI (2017–18) also suggested that the demand for outpatient services is significantly higher than the demand for inpatient care among older adults [5]. Regarding outpatient care, utilization of private facilities was 64%, followed by public facilities (22.7%) and other profit-making healthcare services (like pharmacy/drugstore, home visit, mobile healthcare unit, traditional/folk healers visits, and others) (13.3%) [5].

In developing nations like India, access to private healthcare facilities increases out-of-pocket expenses (OOPE), which traps lower and middle-income people in a vicious cycle of poverty [7]. A previous study suggests that the cost of healthcare services is four times higher in private facilities than in public facilities in India [5, 8]. Similarly, the OOPE is also evidently high when people receive outpatient care from other profit-making healthcare services (like pharmacy/drugstore, home visits, mobile healthcare units, and others) [9]. Apart from the OOPE burden, the quality of treatment is also an issue when people visit other private-profit-making healthcare facilities for outpatient care. The older population primarily depends on households because they are not economically independent in lower-middle-income countries (LMICs), including India [10]. As a result, a substantial number of families experience distress financing when using profit-making healthcare facilities because a considerable number of households belongs to below the poverty line (BPL) and lower and middle socio-economic strata (SES) in India [6, 9, 10]. Health insurance coverage is also deficient in India, particularly among individuals belonging to the lower and middle SES [5, 7, 9]. For instance, only a quarter of households (26%) have health insurance, with 21% registered in the Rashtriya Swasthya Bima Yojana (RSBY), 2.4% in the Central Government Health Scheme, and only 1.4% in private health insurance [5]. However, RSBY does not cover outpatient health care. A significant number of older people (38%) incurred catastrophic health expenditures [5, 11]. At the same time, the increasing trend of private healthcare utilization raises questions about the functionality and quality of care of on-going public health programmes like the National Health Mission (NHM), National Rural Health Mission (NHRM), the Nation Urban Health Mission (NUHM), 3-Tier healthcare system, and National Programme for Healthcare of the Elderly (NPHCE).

Prior studies on the older population and overall healthcare utilization have pointed out that age, sex, educational attainments, wealth status, health insurance, and place of residence are significant determinants of healthcare facilities (private, public, and other profit-making healthcare services) in India [11–15]. However, based on nationally representative data, limited studies have explored the determinants of public or private healthcare utilization for outpatient and inpatient care separately in the Indian context [16, 17]. Evidence from other countries systematically contextualized the determinants of choice and utilization of healthcare facilities for outpatient care [14, 17–22]. Most studies found that age, sex, level of education, morbidity condition, wealth status, health insurance, place of residence, and geographical region significantly determines the choice of outpatient care service among older adults, which varies from country to country [14, 18–21]. In a recent study in Ghana, wealth status and multi-morbidity were found to be significant determinants of both public and private outpatient healthcare service utilization [18]. The choice of healthcare services for outpatient care in China is influenced by several factors, including residence, household income, level of education, health status, and health insurance [14]. The status of chronic illnesses and impairment in older adults were indicators of whether a person would select national and public services for outpatient care in South Korea [19, 20]. In rural South Africa, chronic communicable and non-communicable diseases among older adults are found to be a determining factor in health care utilization for outpatient care [21]. In the context of Burkina Faso, health insurance and the formal occupation of older people are found to be enabling factors of outpatient care utilization [22].

The demand for outpatient care is found to be substantially higher than inpatient care among older adults in India [5], but the existing literature has mainly focused on determinants of healthcare facility choice without separate analysis for outpatient healthcare services [11–17]. Therefore, a study is relevant to define the determinants of public and private healthcare utilization for outpatient care in India. The current paper used the upgraded version of Anderson's behavioral model of healthcare utilization [23, 24] to understand the mechanism of healthcare utilization by selecting explanatory variables for outpatient care in the Indian context. Previous research in Sub-Saharan Africa (SSA) and other countries used the same framework to contextualize the characteristics associated with geriatric healthcare utilization [26–29]. Previous studies have shown that Anderson's specified factors of healthcare facility choice significantly vary with the geographical region, time, demographics, and culture [14, 16–23]. Therefore, applying this model is worthwhile to understand how predictors of healthcare utilization are similar or dissimilar to the existing model used in studies from other countries [14, 16–23]. The present study will assist government stakeholders in revising healthcare programs, strengthening infrastructure, and population choice-based policy making.

## Methods

### Data source

The present study obtained data from the first wave of the Longitudinal Ageing Study in India (LASI 2017–18), conducted by the International Institute for Population Sciences (IIPS) in collaboration with the University of Southern California (USC) and Harvard T.H. Chan School of Public Health (HSPH), and other national and international organisations [5]. The survey was launched with the goal of better understanding the health status of the aging population with socio-economic, demographic, and geographic backgrounds. The sample households in LASI were chosen using a multistage stratified random sampling technique. A three-stage sample approach was adopted for rural areas, while a four-stage sample strategy was used in urban areas. Sample households were chosen from all Indian states and union territories (UTs), except Sikkim, with at least one respondent aged 45 or older [5]. Data was collected using computer-assisted personal interviews (CAPI), which were administered by a professional interviewer [5]. The total sample size for the LASI survey is 72,250 aged 18 years and above [5]. The current study included only 34,588 older individuals (45 years and above) who received outpatient healthcare services in one year prior to the survey in India.

## Variables

### Outcome variable

The outcome variable of the study is the type of healthcare facility accessed for outpatient care. During the survey, interviewers asked a question to the respondents—Which kind of facility did you last visit for outpatient care? The responses were health post/sub-centers, primary health centers, community health centers, district hospital/ sub-district hospital, Government AYUSH hospital, private hospital/nursing home, private clinic (outpatient department (OPD) based service), non-governmental organisations (NGO)/charity/trust/church-run hospital, and private AYUSH hospital, pharmacy/drugstore, home visit, mobile healthcare unit, and others [5]. The AYUSH is an acronym for Ayurveda, Yoga, Naturopathy, Unani, Siddha, and Homeopathy. The outcome variable is categorized into three main categories based on the LASI report—(1) public facilities including health post/sub-centers, primary health centers, community health centers, district hospital/ sub-district hospitals, and government AYUSH hospitals, (2) private facilities comprising private hospital/nursing home, private clinic (OPD based service), NGO/charity/trust/church-run hospital, and private AYUSH hospital, and (3) other facilities including pharmacy/drugstore, home visit, mobile healthcare unit, and others [5, 18]. The rationale behind categorizing the outcome variable is to determine how access to public, private, and other healthcare facilities varies across the socio-demographic characteristics of older adults and states of India.

### Predictor variables

A list of predictor variables was selected using Andersen's healthcare utilization framework model and an extensive literature review based on the Indian context, which includes predisposing factors, enabling factors, and need factors [14, 16–23]. Predisposing factors include demographic parameters like age (45–54 years, 55–64 years, and 65 & above years), sex (male, female), and marital status (never married, currently married, and divorced and others), as well as social-economic factors like years of schooling (no schooling, 1–5 years, 6–11 years, and 12 & above years), and economic activity (no activity, primary, and non-primary activity). Enabling factors are wealth status (poorest, poorer, middle, richer, and richest), health insurance (yes, no), and place of residence (rural, urban). The wealth status was computed based on monthly per capita consumption expenditure [5]. Need factors are the purpose of the visit (immunization, consultation, medical check-up, treatment for illness, and treatment for injury) and self-reported health (Good, fair, and bad). A question, ‘in general, how would you rate your health today?’ was used to assess self-reported health by using a five-point response scale (very good, good, fair, bad, and very bad) [5]. These responses were recorded into three categories namely good (very good/good), fair (fair), and bad (bad/very bad) [18].

### Statistical analysis

The background characteristics of the study population are presented using descriptive statistics with standard errors (SE) in distribution and 95% confidence intervals (CIs). The bivariate analysis was used to show the weighted percentage distribution of utilization of outpatient care by type of healthcare facility (public, private, and other facilities). The details of the sampling weight available in LASI wave 1 report [5]. In bivariate statistics, Pearson's chi-square significance test was performed to evaluate the tests of independence in the distribution. The multinomial logistic regression models were applied to determine the factors affecting the utilization of public and private outpatient care facilities. In multinomial regression models, utilization of other outpatient care facilities is considered as a base outcome or reference category to measure predictor variables linked with public and private outpatient care utilization. In multilevel logistic regression analysis, three sequential models were performed to assess the predictors of utilization of public and private healthcare facilities for outpatient care adopting Andersen's conceptual model [23]. In the current study, only predisposing factors were included as an explanatory variable in the first model, followed by both predisposing and enabling factors in the second model, and all three factors (predisposing, enabling, and need factors) in the final model (Model 3) [18]. The regression results are presented by adjusted odds ratio (AOR) with a 95% confidence interval (CI). All statistical analyses were performed using STATA version 14.0 (StataCorp LP, College Station, TX, USA). The '*svy*' command was used in STATA during the calculation to adjust the effect of the complex survey design (sample weights, strata, and clustering). The maps of the spatial distribution of utilization of public, private, and other outpatient healthcare facilities were presented by the choropleth map in ArcGIS software. Microsoft Excel was used to create the figures for the graphical presentation.

The current study also measured the level of healthcare responsiveness by type of healthcare facilities for outpatient care. The level of healthcare responsiveness is determined based on the following question – ‘Overall, how satisfied were you with your last outpatient visit?’The responses were very satisfied, satisfied, neither satisfied nor dissatisfied, dissatisfied, very dissatisfied [5]. Healthcare responsiveness is categorized as good (very satisfied, satisfied), moderate (neither satisfied nor dissatisfied), and bad (dissatisfied, very dissatisfied) [18].

## Results

### Background characteristics of the study population

Table 1 presents the background characteristics of the study population. More than one-third of the respondents belonged to the age group of 65 years and above (34.8%), followed by 45–54 years (34.3%) and 55–64 years (30.9%). Most respondents were female (56.2%) and belonged to rural areas (64.2%). Most respondents were currently married (73.3%), whereas one-fourth of respondents were currently divorced, separated, or widowed. A substantial number of respondents belonged to the poorest quintile (17.2%) and had no schooling (47.1%). Nearly one-fifth of the respondents had health insurance (23%). More than half of the respondents reported not working (53.5%). The purpose of visits for outpatient care was mainly for illness (69.1%), followed by medical check-ups (19.8%) and consultations (4.1%). The prevalence of poor self-rated health was found to be noteworthy (21.9%).

**Table 1:** Background characteristics of the older adults (aged 45 years and above) who received outpatient care in one year prior to survey in India, LASI wave 1, 2017–18

Variables	n	Percent	SE	95% CI
**Age group**				
45–54 years	11,862	34.3	0.003	33.8–34.8
55–64 years	10,691	30.9	0.002	30.4–31.4
65 & above years	12,035	34.8	0.003	34.3–35.3
**Sex**				
Male	15,146	43.8	0.003	43.3–44.3
Female	19,442	56.2	0.003	55.7–56.7
**Place of resident**				
Rural	22,208	64.2	0.003	63.7–64.7
Urban	12,380	35.8	0.003	35.3–36.3
**Years of schooling**				
No schooling	16,300	47.1	0.003	46.6–47.7
01–05 years	6,440	18.6	0.002	18.2–19.0
06–11 years	8,665	25.1	0.002	24.6–25.5
12 & above years	3,183	9.2	0.002	8.9–9.5
**Marital status**				
Never married	345	1.0	0.001	0.9–1.1
Currently married	25,353	73.3	0.002	72.8–73.8
Divorced and others	8,890	25.7	0.002	25.2–26.2
**Wealth status**				
Poorest	5,933	17.2	0.002	16.8–17.6
Poorer	6,866	19.9	0.002	19.4–20.3
Middle	6,995	20.2	0.002	19.8–20.6
Richer	7,375	21.3	0.002	20.9–21.8
Richest	7,419	21.4	0.002	21.0–21.9
**Health insurance**				
Yes	7,925	22.9	0.002	22.5–23.4
No	26,616	77.0	0.002	76.6–77.5
Missing	47	0.1	-	-
**Economic activity**				
Not working	18,504	53.5	0.003	53.0–54.0
Primary	5,678	16.4	0.002	16.0–16.8
Non-primary	10,406	30.1	0.002	29.6–30.6
**Purpose for visit**				
Immunization	1,302	3.8	0.001	3.6–4.0
Consultation	1,405	4.1	0.001	3.8–4.3
Medical check-up	6,863	19.8	0.002	19.4–20.3
Treatment for illness	23,887	69.1	0.002	68.6–69.6
Treatment for injure	1,131	3.3	0.001	3.1–3.5
**Self-rated health**				
Good	11,332	33.1	0.003	32.6–33.6
Fair	15,316	44.8	0.003	44.2–45.3
Bad	7,567	22.1	0.002	21.7–22.6
Missing	371	1.1	-	-
** Total (n)**	**34,588**	**100**	**-**	**-**

**Note:** n refers sample size, SE stands Standard Errors, and CI refers Confidence Intervals

### Distribution of utilization of healthcare facilities by older adults in India

**Fig. 1:**
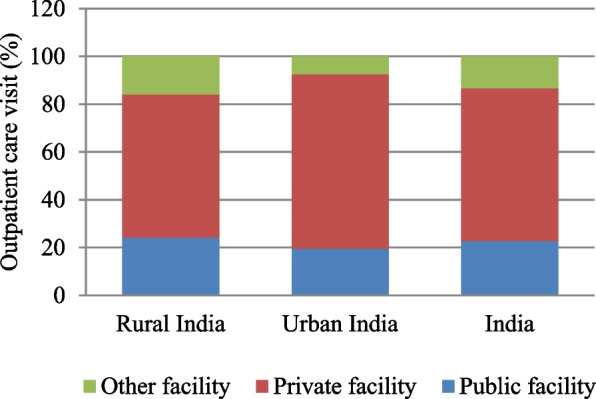
Distribution of outpatient care utilization by type of health facilities among older (aged 45 years and above), India, LASI wave 1, 2017–18

**Fig. 2:**
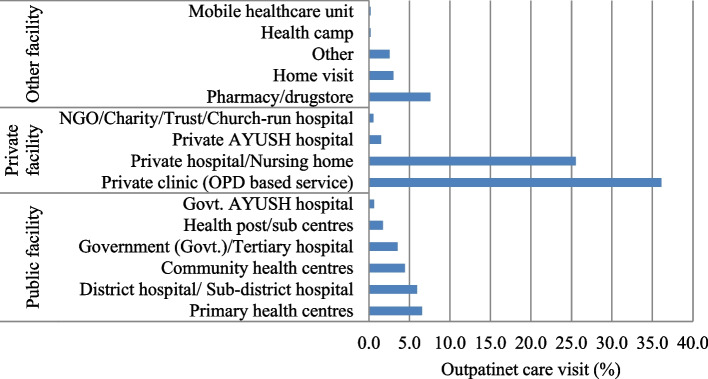
Percent distribution of outpatient care utilization by type of health units among older (aged 45 years and above), India, LASI wave 1, 2017–18

In India, older people most commonly used private facilities (63.7%), followed by public facilities (22.8%), and other facilities (13.5%) for their outpatient care. Furthermore, a slight variation in utilization of public and private healthcare except for other providers is observed between rural and urban India (Fig. 1). The utilization of private clinics (OPD-based service) (36.1%) and private hospitals/nursing homes (25.5%) was seen to be considerably high as compared to other private healthcare facilities (such as private AYUSH hospital, and NGO/charity/trust/church-run hospital) (Fig. 2). Among the public healthcare facilities, 6.6 percent of older adults accessed primary health centers (PHCs), followed by district hospitals/sub-district hospitals (5.9%) and community health centers (CHCs) (4.4%). In other facilities, the use of pharmacies and drugstores (7.6%) was noteworthy.

### Levels and patterns of utilization of public, private, and other healthcare facilities by background characteristics

There was significant variation in the utilization of public, private, and other healthcare facilities for outpatient care across the categories of age, sex, and marital status (Table 2). The use of public outpatient care was significantly higher among older adults with a lower level of education and belonged to the poorest quintile as compared to their counterparts respectively. There was a significant gap in the utilization of public healthcare facilities between the health insured (30.2%) and non-insured (20.5%) groups. The utilization of public healthcare facilities for immunization (37.4%) and treatment of injuries (29.6%) was noteworthy. The use of private healthcare facilities was 10% higher among older adults with 12 or more years of schooling (76.9%) than those with no schooling (59.9%). A similar tendency was seen with increasing wealth quintiles. Older adults favored private healthcare facilities for their medical check-ups (71.4%), followed by consultations (66.5%), and illness treatment (62.9%).The utilization of other healthcare services was considerably higher among the older adults who belong to the poorest wealth quintile (19.4%) and rural areas (16.2%), who are never married (16.7%), and those without schooling (16.4%) and without health insurance (14.9%) as compared to their respective counterparts.

**Table 2:** Levels and patterns of utilization of public, private, and other healthcare facilities for outpatient care by background characteristics of older adults (aged 45 years and above) in India, LASI wave 1, 2017–18

	**Public facility [**95% CI]	**Privet facility [**95% CI]	**Others [**95% CI]	**Chi-square *p* value**
**Age group**				*p* ≥ 0.129
45–54 years	22.9 [22.6–23.2]	64.2 [64.1–64.4]	12.9 [12.7–13.2]	
55–64 years	22.3 [21.9–22.6]	63.5 [63.4–63.8]	14.2 [14.1–14.6]	
65 & above years	23.0 [22.7–23.2]	63.4 [63.1–63.7]	13.5 [13.3–13.6]	
**Sex**				*p* ≤ 0.001
Male	23.5 [2.3.1–23.6]	62.7 [62.4–62.9]	13.8 [13.4–13.9]	
Female	22.2 [22.0–22.4]	64.5 [64.3–64.7]	13.3 [13.0–13.5]	
**Place of resident**				*p* ≤ 0.001
Rural	24.1 [23.8–24.3]	59.8 [59.7–60.0]	16.2 [16.1–16.5]	
Urban	19.8 [19.7–20.0]	72.8 [72.6–72.9]	7.4 [7.0–7.6]	
**Years of Schooling**				*p* ≤ 0.001
No schooling	23.8 [23.4–24.3]	59.9 [59.7–60.1]	16.4 [16.2–16.5]	
01–05 years	26.4 [26.3–26.8]	61.7 [61.5–62.0]	11.9 [11.7–12.1]	
06–11 years	20.8 [20.6–20.9]	69.3 [69.1–69.6]	9.9 [9.8–10.2]	
12 & above years	14.0 [13.7–14.2]	76.9 [76.8–77.0]	9.1 [8.7–9.2]	
**Marital status**				*p* ≤ 0.001
Never married	25.7 [25.5–25.9]	57.6 [57.4–57.7]	16.7 [16.6–16.9]	
Currently married	21.8 [21.3–22.6]	65.0 [64.7–65.2]	13.2 [13.0–13.5]	
Divorced and others	25.3 [24.7–25.9]	60.3 [60.1–60.6]	14.4 [14.3–14.8]	
**Wealth status**				*p* ≤ 0.001
Poorest	27.5 [27.3–27.8]	53.1 [52.7–53.5]	19.4 [19.3–19.5]	
Poorer	23.9 [23.8–24.2]	60.9 [60.8–61.0]	15.3 [15.1–15.6]	
Middle	22.7 [22.4–23.1]	64.0 [63.7–64.2]	13.3 [13.2–13.7]	
Richer	21.3 [21.2.21.5]	67.5 [67.4–67.7]	11.1 [11.0–11.3]	
Richest	18.5 [18.4–18.7]	72.8 [72.7–73.0]	8.8 [8.7–9.0]	
**Health insurance**				*p* ≤ 0.001
Yes	31.2 [30.4–31.5]	60.3 [60.2–60.5]	8.5 [8.1–8.7]	
No	20.5 [20.3–20.4]	64.6 [64.3–64.8]	14.9 [14.7–15.0]	
**Economic activity**				*p* ≤ 0.001
Not working	22.4 [22.1–22.7]	64.5 [64.4–64.7]	13.1 [13.0–13.4]	
Primary	24.6 [24.3–24.9]	59.5 [59.1–60.0]	15.9 [15.7–16.2]	
Non-primary	22.0 [21.9–22.1]	65.3 [65.1–65.8]	12.7 [12.4–12.8]	
**Purpose for visit**				*p* ≤ 0.001
Immunization	37.4 [37.2–37.7]	49.0 [48.9–49.3]	13.6 [13.3–13.7]	
Consultation	25.2 [25.0–25.7]	66.5 [66.3–66.8]	8.3 [8.2–8.5]	
Medical check-up	24.1 [23.8–24.3]	71.4 [71..3–71.6]	4.5 [4.1–4.6]	
Treatment for illness	21.2 [21.0–21.4]	62.9 [62.7–63.2]	16.0 [15.8–16.4]	
Treatment for injure	29.6 [29.3–29.8]	59.3 [59.1–59.4]	11.1 [11.0–11.3]	
**Self-rated health**				*p* ≤ 0.001
Good	21.5 [21.4–21.7]	64.1 [63.7–64.6]	14.4 [14.3–14.5]	
Fair	22.6 [22.4–22.9]	64.1 [64.0–64.3]	13.3 [12.9–13.7]	
Bad	25.1 [24.7–25.3]	62.5 [62.3–62.6]	12.5 [12.2–13.0]	
** Over all**	**22.8 [22.5–23.1]**	**63.7 [63.5–63.8]**	**13.5 [13.4–13.7]**	**-**

### Spatial patterns of utilization of public, private, and other healthcare facilities

**Fig. 3:**
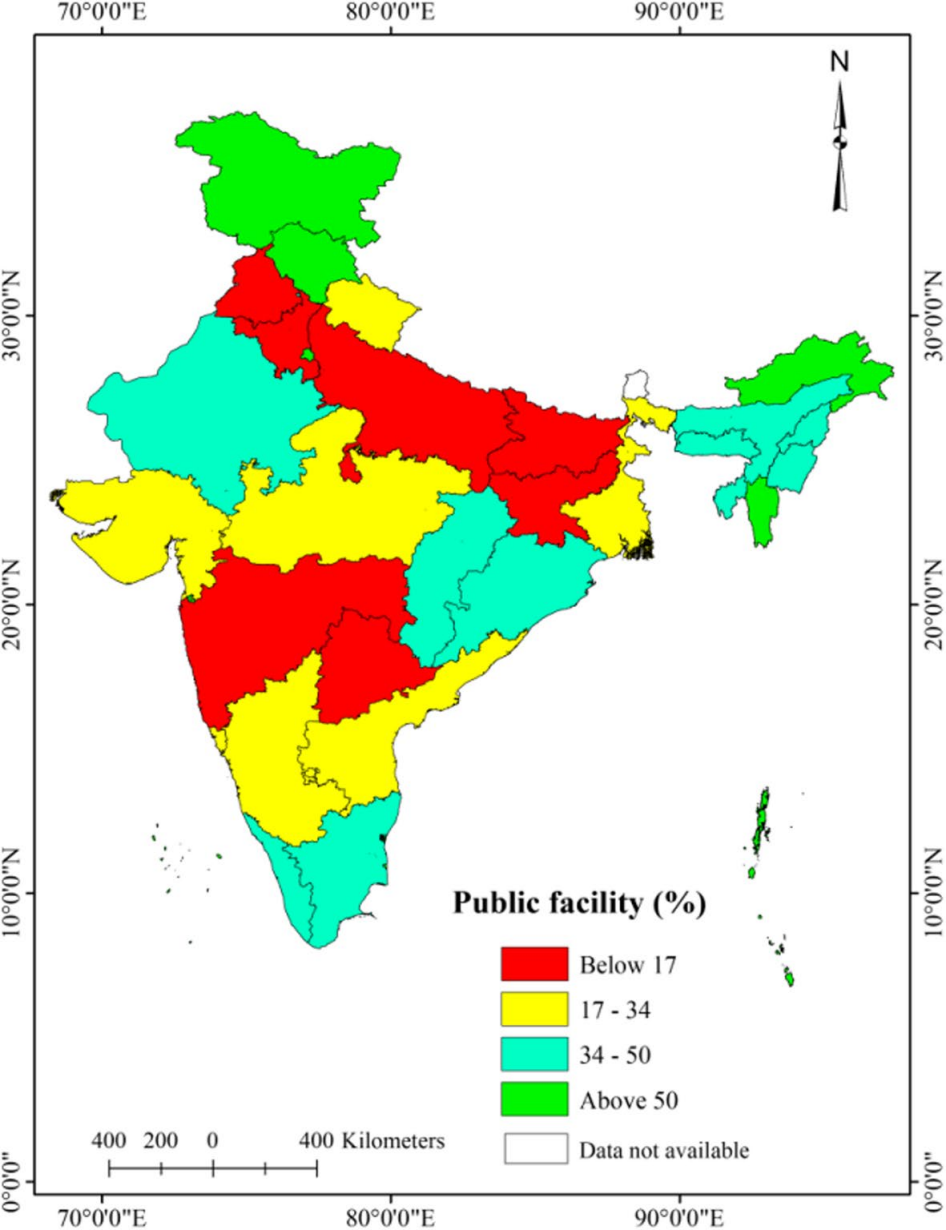
Spatial patterns of public outpatient healthcare utilization, older adults (aged 45 years and above), India, LASI wave 1, 2017–18

**Fig. 4:**
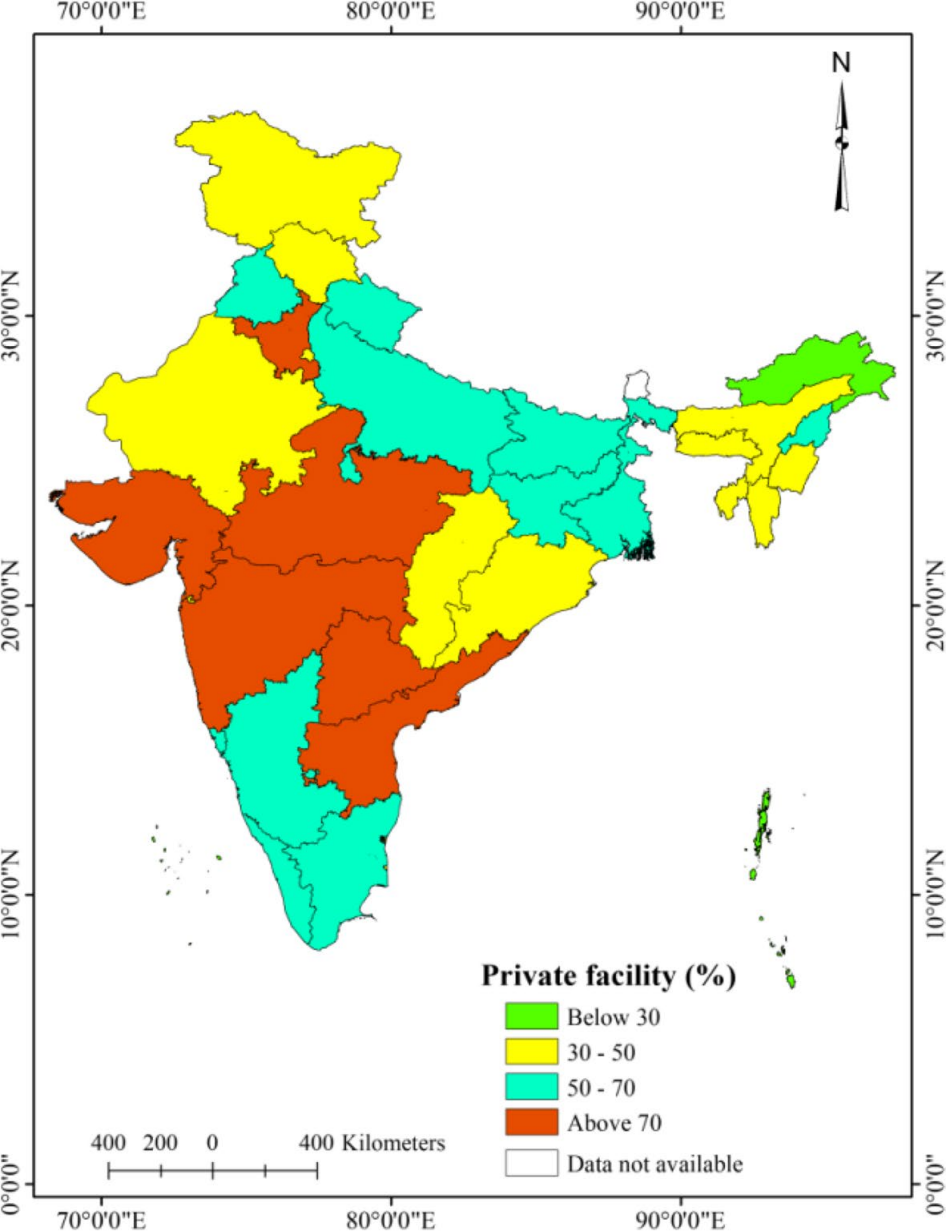
Spatial patterns of private outpatient healthcare utilization, older adults (aged 45 years and above), India, LASI wave 1, 2017–18

**Fig. 5:**
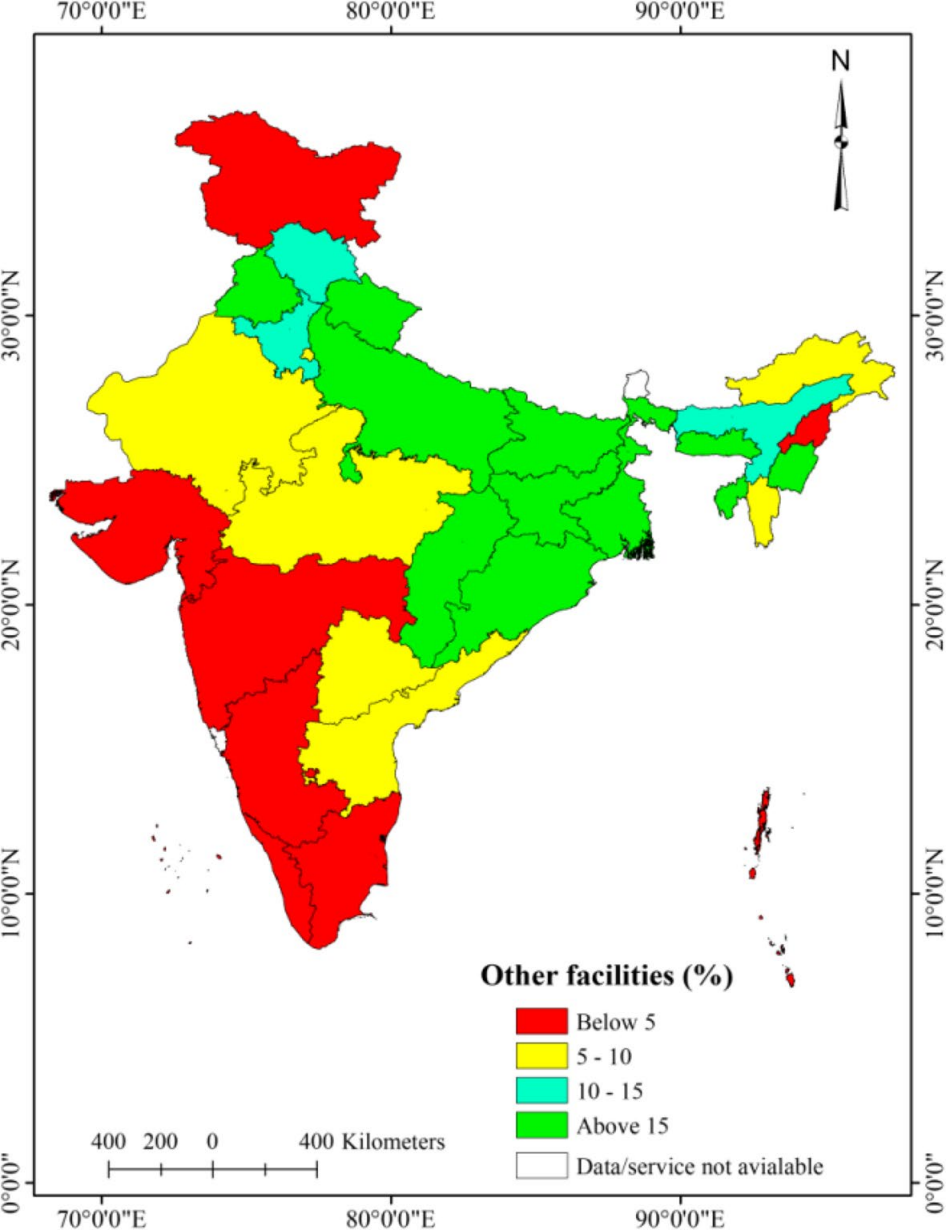
Spatial patterns of utilization of other facility for outpatient, older adults (aged 45 years and above), India, LASI wave 1, 2017–18

The level of utilization of private facilities was noticeably higher in the majority of the states in India, except in the North-eastern hilly states as compared to the public and other healthcare facilities (Fig. 3 and 4). A lower level of utilization of public health facilities was observed in Bihar (10.3%), Telangana (10.8%), Maharashtra (12.2%), Uttar Pradesh (13.7%), and Jharkhand (14.1%) (Fig. 3). The utilization of other healthcare facilities was considerably higher in Bihar (30.8%), Meghalaya (27.4%), Tripura (26.5%), Punjab (25.7%), Uttar Pradesh (24.5%), Manipur (19.8%), Jharkhand (19.7%) and West Bengal (19.4%) than the rest of the states of India (Fig. 5).

### Factors influencing the utilization of public healthcare facilities

The level of education (years of schooling) was the most significant predisposing factor for the utilization of public healthcare facilities. The likelihood of utilization of public healthcare facilities was 25% higher among older adults with 6–11 years of schooling (AOR: 1.25; 95% CI: 1.13–1.39) compared to those with no schooling. Place of residence, wealth status, and health insurance coverage were also found to enabling factors for utilization of public outpatient healthcare. Older adults who belonged to urban areas were 93% more likely to utilize public facilities (AOR: 1.93; 95% CI: 1.76–2.12) than their rural counterparts. Compared to the poorest quintile, the richest quintile was 1.53 times more likely to utilize public facilities (AOR: 1.53; 95% CI: 1.35–1.72). The older adults who had no health insurance were 46% less likely to utilize public healthcare facilities (AOR: 0.54; 95% CI: 0.49–0.59) than their counterparts. The purpose of health visits and self-rated health were both significant need factors for utilization of public healthcare facilities. Older adults who had sought treatment for illness were 50% less likely to utilize public healthcare facilities (AOR: 0.50; 95% CI: 0.41–0.60) compared to those who visited for immunization.

### Factors influencing the utilization of private healthcare facilities

Sex and years of schooling were the significant predisposing factors for the utilization of private healthcare facilities (Table 3, Model 3). Females were 18% more likely to access private facilities (AOR: 1.18; 95% CI: 1.09–1.28) than males. Older adults who had completed 12 or more years of schooling were 45% more likely to use private facilities (AOR: 1.45; 95% CI: 1.24–1.69) than those with no education. Place of residence, wealth status, and health insurance coverage were also significant enabling factors for utilization of private healthcare facilities. In urban areas, the likelihood of using private facilities was two times more likely (AOR: 2.06; 95% CI: 1.89–2.24) than in rural counterparts. The older adults in the richest quintile were 2.7 times more likely (AOR: 2.7; 95% CI: 2.41–3.02) to use private healthcare facilities than their poorest counterparts. Compared to the health-insured group, the older adults with no health insurance were 28% less likely (AOR: 0.72; 95% CI: 0.66–0.79) to utilize private facilities. Purpose of health visits and self-rated health were also found as need factors for utilizing private healthcare facilities. In particular, older adults preferred twice the private facilities for medical check-ups (AOR: 2.09; 95% CI: 1.68–2.6) to those who visited for vaccination. Further, older individuals with poor self-rated health status were 1.3 times more likely (AOR: 1.33; 95% CI: 1.21–1.47) to visit private healthcare facilities than those who reported their good self-rated health.

**Table 3:** Multinomial logistic regression models assessing the factors associated with public and private outpatient healthcare utilization in India, older adults (aged 45 years and above), LASI wave 1, 2017–18

	**Model 1**		**Model 2**		**Model 3**	
	**Public (1) vs. Others (0)**	**Private (1) vs. Others (0)**	**Public (1) vs. Others (0)**	**Private (1) vs. Others (0)**	**Public (1) vs. Others (0)**	**Private (1) vs. Others (0)**
	AOR (95% CI)	AOR (95% CI)	AOR (95% CI)	AOR (95% CI)	AOR (95% CI)	AOR (95% CI)
**Age group**						
45–54 years ®	1.00	1.00	1.00	1.00	1.00	1.00
55–64 years	0.99 (0.91–1.09)	1.10 (1.01–1.19)	0.97 (0.89–1.06)	1.08 (0.99–1.17)	0.93 (0.85–1.02)	1.04 (0.96–1.14)
65 & above years	1.04 (0.95–1.13)	1.11 (1.02–1.2)	0.98 (0.89–1.07)	1.05 (0.96–1.15)	0.90 (0.82–0.99)	0.98 (0.90–1.07)
**Sex**						
Male ®	1.00	1.00	1.00	1.00	1.00	1.00
Female	**1.19 (1.1–1.28)**	**1.39 (1.29–1.49)**	1.03 (0.94–1.12)	**1.20 (1.11–1.29)**	1.02 (0.94–1.11)	**1.18 (1.09–1.28)**
**Years of Schooling**						
No schooling ®	1.00	1.00	1.00	1.00	1.00	1.00
01–05 years	**1.68 (1.53–1.86)**	**1.53 (1.39–1.68)**	**1.41 (1.28–1.56)**	**1.26 (1.15–1.39)**	**1.34 (1.21–1.49)**	**1.21 (1.10–1.34)**
06–11 years	**1.79 (1.62–1.97)**	**2.00 (1.83–2.18)**	**1.33 (1.2–1.47)**	**1.39 (1.26–1.53)**	**1.25 (1.13–1.39)**	**1.32 (1.20–1.46)**
12 & above years	**1.61 (1.38–1.88)**	**2.83 (2.45–3.25)**	0.98 (0.83–1.16)	**1.51 (1.30–1.76)**	**0.92 (0.78–1.09)**	**1.45 (1.24–1.69)**
**Place of resident**						
Rural ®			1.00	1.00	1.00	1.00
Urban			**2.11 (1.93–2.31)**	**2.20 (2.02–2.39)**	**1.93 (1.76–2.12)**	**2.06 (1.89–2.24)**
**Wealth status**						
Poorest ®			1.00	1.00	1.00	1.00
Poorer			1.10 (0.99–1.23)	**1.41 (1.28–1.56)**	1.1 (0.99–1.23)	**1.41 (1.28–1.56)**
Middle			**1.3 (1.17–1.46)**	**1.87 (1.69–2.08)**	**1.29 (1.15–1.44)**	**1.85 (1.67–2.06)**
Richer			**1.37 (1.22–1.53)**	**2.22 (1.99–2.47)**	**1.32 (1.18–1.48)**	**2.16 (1.94–2.40)**
Richest			**1.66 (1.47–1.87)**	**2.88 (2.57–3.23)**	**1.53 (1.35–1.72)**	**2.7 (2.41–3.02)**
**Health insurance**						
Yes ®			1.00	1.00	1.00	1.00
No			**0.51 (0.47–0.56)**	**0.69 (0.63–0.76)**	**0.54 (0.49–0.59)**	**0.72 (0.66–0.79)**
**Economic activity**						
Not working ®			1.00	1.00	1.00	1.00
Primary			0.96 (0.86–1.06)	1.03 (0.93–1.14)	1.01 (0.91–1.13)	1.08 (0.98–1.20)
Non-primary			0.82 (0.75–1.90)	0.87 (0.80–1.25)	0.87 (0.79–1.10)	0.91 (0.83–1.09)
**Purpose for visit**						
Immunization ®					1.00	1.00
Consultation					1.01 (0.76–1.34)	**1.46 (1.11–1.92)**
Medical check-up					**1.64 (1.31–2.04)**	**2.09 (1.68–2.60)**
Treatment for illness					**0.50 (0.41–0.60)**	0.82 (0.68–0.99)
Treatment for injure					0.97 (0.73–1.29)	1.18 (0.89–1.55)
**Self-rated health**						
Good ®					1.00	1.00
Fair					**1.12 (1.04–1.22)**	**1.18 (1.09–1.27)**
Bad					**1.38 (1.24–1.53)**	**1.33 (1.21–1.47)**

Note: *AOR* refers adjusted odds ratio, *CI* stands Confidence Intervals; ® refers reference category;others includes pharmacy/drugstore, home visit, mobile healthcare unit, and others; All bold results are statistically significant at level of significance *p* ≤ 0.010

### Healthcare responsiveness by type of healthcare facilities

**Fig. 6:**
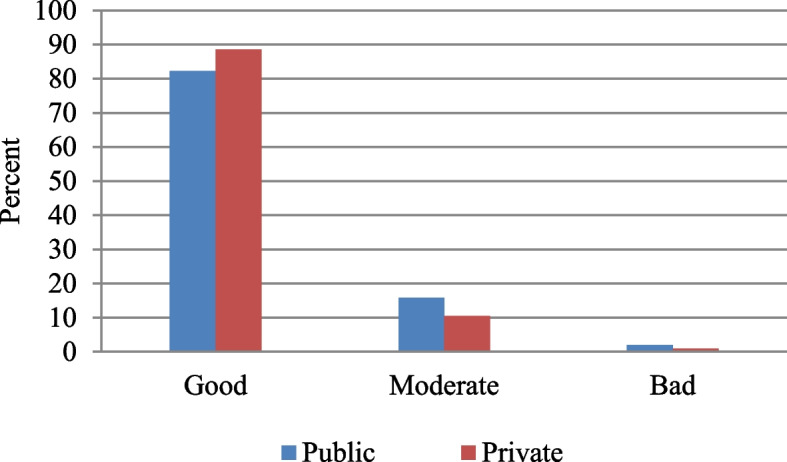
Level of healthcare responsiveness by type of healthcare facility for outpatient care among older adults (aged 45 years and above) in India, LASI wave 1, 2017–18

More than 80% of older adults reported that they were highly satisfied (rated as good) with both public and private outpatient services (Fig. 6). However, the satisfaction level was found to be slightly higher in private facilities (89%) than in public facilities (82%). At the same time, the level of poor satisfaction in healthcare facilities is twice as in public facilities (2%) than in private facilities (1%).

## Discussion

The current study endeavors to examine the patterns and determining factors of the utilization of healthcare for outpatient care among older adults in India. The results demonstrated that the utilization of private health facilities was more than double of public health facilities in India. The result is consistent with previous findings in India and South Korea [12, 16, 17, 20] and inconsistent with findings from Ghana, China, and Burkina Faso [14, 18, 22]. The majority of the studies highlighted that the public sector is grappling with the shortage of skilled doctors and nurses, inadequate infrastructure and poor quality of services, and so on in the Indian context [9, 12, 24], which eventually leads to high dependency on the private sector [9, 13].

The present study found year of schooling is a significant predisposing factor for utilization of both public and private healthcare facilities. The result is similar to many previous studies [16, 18]. The likelihood of utilization of healthcare facilities increases (i.e., public and private facilities vs. other facilities) with increasing years of schooling found in the current study. The similar findings were also noted in the previous studies in India [12, 13, 16] and elsewhere [18, 20]. Some plausible explanations related to the positive association between public/private healthcare facility utilization and levels of education are the following: First, older adults with greater educational attainment are better informed and tend to understand available information regarding medical treatment [24]. Second, education is a mediator of healthcare choice, which increases the ability to determine the best health services [33]. Finally, more education is meant to lead to improved professional opportunities and high financial and social status [17, 24]. As a result, educational attainment is associated with the choice of healthcare facilities.

In line with previous studies in India [11–13] and elsewhere [18, 20, 22], wealth status, health insurance, and the place of residence were found to be important enabling factors of utilization of both private and private health facilities among the older population. The likelihood of using both public and private healthcare facilities increases with increasing wealth status. Economic wellness promotes affordability and enables older individuals to bear the expenses both in the private and public sectors [26]. A study from Nepal revealed that health-seeking behaviour is strongly associated with financial status [27]. The utilization of private healthcare facilities is higher among the wealthier sections of society, while the utilization of public healthcare facilities is prevalent among both the richest and poorest groups. Since insured people are more aware of the benefits of regular health check-ups and are financially secure, they enroll themselves in either publicly or privately financed insurance schemes to avoid large out-of-pocket payments [11, 17]. Besides, the extent of utilization of healthcare facilities varies by a large extent by place of residence specifically owing to accessibility and availability. Prior studies in India have also documented a significant gap between the availability of public and private providers in terms of place of residence since urban dwellers have the choice of both public and private facilities, while rural dwellers often rely on public facilities [13]. Another study from India [15] unfolded the barriers to accessing healthcare facilities in a rural area over the urban area, as only one-third (37%) of rural dwellers have accesses to healthcare facilities within a 5 km radius of their respective location.

The purpose of the visit and self-rated health status was identified as important need factors in healthcare choice for outpatient treatment in this study. The outcome is consistent with earlier piece of research in China [14]. In India, older adults mostly preferred private facilities compared to other facilities, especially for consultation and medical check-ups. It may be mainly due to quality health care services and the aversion of the patient crowd in public facilities [9]. Older adults with poor self-rated health are more likely to utilize both private and public facilities than other healthcare facilities. This finding is consistent with previous research works in China [14]. Older people with poor health status are more prone to frequent routine check-ups, and are thus compelled to move to outpatient care in the absence of caregivers to avoid spending the night in hospitals [14].

The current study discovered that a considerable percentage of older persons from lower SES (mainly belonging to a lower level of education and income) also received outpatient care at private facilities in India. Earlier studies have demonstrated that higher use of private healthcare facilities among individuals belonging to lower and middle SES socioeconomically disadvantaged populations is linked with long-term poverty in India and China [28]. Furthermore, individuals with lower-level of education are less aware of specialization of care providers, treatment billing, and other costs, which could put them at risk of receiving substandard health care, over charging for medical care, over medication, and fraud [24]. Few other studies corroborate the same and revealed that private sector utilization pushes uninsured older people into poverty in absence of financial security coverage [28, 29]. Although several health schemes, such as the Ayushman Bharat Pradhan Mantri Jan Arogya Yojana (ABPM-JAY), the Employees State Insurance Scheme (ESI), and the Central Government Health Scheme (CGHS), are effectively paying healthcare costs, India is regarded as one of the highest contributing countries in out-of-pocket expenditure (63%) to total health care expenditure globally [24,37]. The ABPM-JAY provides coverage of 500,000 per family/annum covering around 50 crores beneficiaries till 2019, which may help pave the way for achieving universal health coverage (UHC) in the near future [5, 29].

With India's expanding ageing population, the UHC is becoming an emerging public health concern due to ageing-related health problems such as frailty and chronic morbidities [5]. In this context, the Government of India has already launched a variety of health programmes including NHM, NRHM, NUHM, NPHCE promote universal health coverage [9]. Still the utilization of other facilities for outpatient care is noticeable in India, particularly in Bihar, Meghalaya, Tripura, Punjab, Uttar Pradesh, Manipur, and Jharkhand found in the present study. At the same time, the increasing trend of private healthcare utilization raise questions on functionally and quality of care of the on-going National Programme for Healthcare of the Elderly (NPHCE) in India.

### Implications

The current study accounts for several policy implications. Firstly, the higher utilization of private outpatient care in India is found in the present study. The government needs to focus on private organizations to provide treatment at affordable prices to patients. In addition, the promotion of public–private partnerships is also welcome to build a sustainable healthcare system. Secondly, there is an urgent need to strengthen public health infrastructure for ensuring quality and efficient healthcare services, particularly in the states with lower utilization of public healthcare facilities. Thirdly, the present study found a significant spatial disparity in the distribution of healthcare utilization, suggesting implementations of healthcare policy and programmes should be formulated using a spatial-equity healthcare services approach. Fourthly, the areas with high use of other facilities (Eastern India, including Uttar Pradesh and the Northeast hilly region) where need to introduce outpatient care training programme for other healthcare providers (pharmacists, quack doctors, and others). Lastly, there is an urgent need to promote coverage of all public and private health insurance schemes covering outpatient care. Moreover, it is necessary to cover all parsons specifically those who belong to the lower and middle SES.

### Limitations and strengths

The present study is subjected to several shortcomings. Firstly, the findings are prone to recall bias as the study was based on self-reported responses. Secondly, the current study is unable to capture a causal association between outcome and predictor variables owing to the cross-sectional nature of the data. Thirdly, the sectors of healthcare facilities may be varied from one visit to another. With this current nature of data, it was not possible to capture this variation in the utilization of healthcare facilities. Therefore, future longitudinal research can be undertaken to determine the causality between the utilization of outpatient healthcare facilities and associated predictors.

However, the present study is worthwhile because this study provides several insights into the body literature. Firstly, the study has systematically documented regional heterogeneity in the utilization of outpatient care by type of health facilities in the Indian context. Secondly, the findings can be generalized to all Indian older adults since the study is based on a national sample survey. Thirdly, the outcomes of the research revealed the current poor-rich gap in the utilization of outpatient healthcare facilities, as affluent older persons benefited from both public and private healthcare facilities. Fourthly, our findings have also revealed the extent of the gap between public and private healthcare.

## Conclusion

To conclude, the foremost challenge India faces is an increase in the number of people with more specialized healthcare demands with the expanding ageing population. In this regard, public healthcare services, which seem to be the foundation of universal healthcare coverage, are often underutilized, owing to poor quality of care, and inadequate infrastructure, causing private healthcare to become the major source of healthcare services. Consequently, marginalized and poorest of the elderly are either forced to depend on profit-driven private healthcare institutions or seek other healthcare options (like pharmacy/drugstore, quacks, and others). In this setting, heavy dependence on private facilities in the absence of any insurance coverage often pushes marginalized older adults to the verge of destitution. Therefore, government intervention is crucially important in controlling the costs of private for-profit facilities to transform them into the pro-people, accessible, and affordable health services that they were intended to be. Moreover, improving outpatient care through an increasing number of trained health care providers, quality assurance both in clinical and non-clinical domains such as less waiting time, and neat and user-friendly outdoor setup in publicly funded health facilities would be an imperative step toward achieving the goal of universal health coverage and reducing health care related financial risk as well.

## Data Availability

The LASI Wave-1 data was collected by the nodal institution International Institute for Population Sciences, Mumbai on behalf of the Ministry of Health and Family Welfare, Government of India. All data were de-identified. The de-identified version of the LASI Wave-1 data is publicly available to the researchers and policymakers upon formal request to the International Institute For Population Sciences for access (link to the data request document LASI_DataRequestForm_0.pdf (iipsindia.ac.in) and link for the other information for lasi data set LASI Wave-I | International Institute for Population Sciences (IIPS) (iipsindia.ac.in).

## Abbreviations

AOR: Odds ratio
OR: Odds ratio
LASI: Longitudinal Ageing Study in India
CAPI: Computer-Assisted Personal Interview
CI: Confidence interval
SES: Socio-economic strata
IIPS: International Institute for Population Sciences
USC: University of Southern California
HSPH: Harvard T.H. Chan School of Public Health
AYUSH: Ayurveda, Yoga, Naturopathy, Unani, Siddha, and Homeopathy
ABPM-JAY: Ayushman Bharat Pradhan Mantri Jan Arogya Yojana
ESI: Employees State Insurance Scheme
CGHS: Central Government Health Scheme
RSBY: Rashtriya Swasthya Bima Yojana
BPL: Below Poverty Line
CDs: Communicable diseases
NCDs: Non-communicable diseases
CVDs: Cardio-vascular-diseases
OOPE: Out-of-pocket expenses
